# Knowledge, attitude and practice on continuous glucose monitoring and self-monitoring of blood glucose among healthcare professionals across India: A cross-sectional study

**DOI:** 10.17925/EE.2026.22.2.7

**Published:** 2026-10-20

**Authors:** Nitin Kapoor, Shehla Sheikh, Sambit Das, Navneet Agarwal, Mukut Roy, Sanjay Kalra

**Affiliations:** 1. Department of Endocrine, Diabetes & Metabolism, Christian Medical College, Vellore, India; 2. Department of Endocrinology, KGN Diabetes and Endocrine Center, Mumbai, India; 3. Department of Endocrinology, Kalinga Institute of Medical Sciences, Bhubaneswar, India; 4. Department of Endocrinology, Diabetes, Obesity & Thyroid Center, Gwalior, India; 5. Department of Endocrinology, Agartala Government Medical College & GBP Hospital, Agartala, India; 6. Department of Endocrinology, Bharti Hospital, Karnal, India

**Keywords:** Clinical practice, continuous glucose monitoring, diabetes management, diabetes mellitus, glycemic variability, lifestyle, self-monitoring of blood glucose

## Abstract

**Background:**

Uncontrolled glycemia remains a major concern in India, underlining the need for wider adoption of effective glucose monitoring methods in diabetes care. Despite the benefits of continuous glucose monitoring (CGM), its adoption remains low in India and is unevenly distributed across healthcare settings and patient populations.

**Methods:**

A cross-sectional, questionnaire-based survey was conducted among healthcare professionals (HCPs) practicing across private and public healthcare settings in India to assess their knowledge, attitudes and practices regarding CGM metrics using an 18-item questionnaire. Responses were measured using a 5-point Likert scale.

**Results:**

A total of 126 HCPs from different specialties participated in the study. Most HCPs (81.7%) expressed confidence in the use of CGM in patients with gestational diabetes mellitus. The majority of HCPs were confident or highly confident in interpreting CGM reports (80.1%) and explaining them to patients (81.8%). More than 90% of HCPs agreed that CGM improves overall diabetes management and 88.1% were confident that CGM helps reduce hypoglycemic and hyperglycemic episodes compared with self-monitoring of blood glucose (SMBG) alone. Most HCPs (78.6%) agreed that the cost of CGM is a major barrier to its wider adoption in diabetes care. Despite recognizing the clinical value of CGM, fewer than half of HCPs believed it could completely replace SMBG.

**Conclusion:**

The study highlights the adoption–action gap of CGM use in diabetes care in Indian settings; however, the predominantly experienced, private-sector sample may limit generalizability to the broader HCP population. High costs, variable confidence in CGM interpretation and continued reliance on SMBG contributed to the gap despite HCPs’ strong awareness of CGM’s clinical value. Addressing these barriers and improving training may enhance CGM adoption nationwide.

Diabetes mellitus, a multifactorial, chronic and progressive metabolic disorder primarily caused by elevated blood glucose levels, is a growing global health epidemic and a leading cause of adult mortality.^1,2^ By 2030, global diabetes prevalence is estimated to rise to 7,079 per 100,000 individuals, and by 2040, to 8,862.^3,4^ Type 2 diabetes mellitus (T2DM), characterized by insulin resistance and hyperglycemia, is a serious public health concern affecting an estimated 462 million people (6.28% of the population) worldwide in 2017 and is associated with a reduced quality of life among patients and high healthcare expenditures.^3–5^ The prevalence of diabetes has been rising more rapidly in low- and middle-income countries (LMICs) than in high-income countries.^2^ India bears one of the largest burdens of diabetes, with the prevalence in adults being 10.5%.^6^ There has been an exponential growth in the prevalence of T2DM in India over the past few decades, rising from 5.0% in 1980 to 8.8% in 2014.^7^

Uncontrolled glycemia remains a major concern in India, underlining the need for nationwide efforts to adopt and implement effective glucose monitoring methods for optimal diabetes management.^1,8,9^ Commonly available glucose monitoring approaches include glycated hemoglobin (HbA1c) measurement, self-monitoring of blood glucose (SMBG) and continuous glucose monitoring (CGM).^8,9^ Although SMBG is considered the gold standard for assessing glycemic status, it cannot provide real-time data on blood glucose levels for an entire day, cannot detect asymptomatic hypoglycemia and has limited capacity for long-term glycemic tracking.^8,10^ In contrast, CGM devices use minimally invasive skin sensors to continuously measure interstitial glucose and provide a comprehensive, real-time evaluation of glucose patterns and dynamic changes in glucose levels in patients with type 1 diabetes mellitus (T1DM), patients with T2DM treated with insulin, patients with gestational diabetes mellitus (GDM), pediatric patients with diabetes and even individuals with prediabetes.^8,10,11^ Moreover, compared with conventional markers such as HbA1c, CGM offers additional metrics for glucose management, including time in range (TIR), time above range (TAR), time below range (TBR) and glycemic variability (GV).^1^ Additionally, CGM provides key behavioral insights that help healthcare professionals (HCPs) make informed decisions and manage diabetes effectively.^1,12^

Despite these benefits, adoption of CGM remains low in India and is unevenly distributed across healthcare settings and patient populations. It is largely limited to a small, high-risk group, primarily patients with T1DM or patients with T2DM on intensive insulin therapy, and is minimal among patients with noninsulin-treated T2DM.^12^ High costs, limited availability, inconsistent adoption, resource constraints within the healthcare system and limited access to particular patient populations are major challenges associated with CGM use in India.^13^ HCPs from different specialties involved in diabetes management play an essential role in the appropriate adoption and use of diabetes technologies. Assessing their knowledge, attitudes and practices (KAP) can help identify gaps and guide strategies for appropriate CGM use in clinical practice. However, there is a lack of data from India on HCPs’ knowledge and attitudes about CGM and its use in clinical practice for diabetes care.

The present survey-based study aimed to evaluate the KAP of HCPs in India regarding CGM metrics; assess the implementation of CGM in clinical practice; examine the perceived role of SMBG in the era of CGM and identify key barriers to CGM adoption in India.

## Materials and methods

### Survey design and setting

A cross-sectional, questionnaire-based survey was conducted between November 2025 and February 2026 among HCPs practicing in both private and public healthcare settings across multiple geographic locations in India. The survey was collaboratively designed by the authors in consultation with an expert review panel comprising specialists in diabetes, endocrinology and internal medicine. The survey was conducted via Google Forms (Google, Mountain View, CA, USA) and it took approximately 30 minutes to complete.

### Participant recruitment

A total of 150 HCPs were invited to participate in the study, with 30 physicians invited from each of the 5 geographical regions (East, West, North, South and Central) of India. Participants were purposively identified through online medical panels and among attendees of a 2025 medical conference on CGM in India, based on their clinical specialties and involvement in diabetes care. HCPs were invited via secure email to complete a screening questionnaire to determine their eligibility. Eligible HCPs were then provided with a password-protected survey link to complete an online, self-administered survey. Eligible participants were adult medical practitioners in India from multiple specialties, including diabetologists, endocrinologists, cardiologists, ophthalmologists, pediatricians and internal medicine specialists with ≥1 year of clinical practice experience who had managed patients with diabetes within the preceding 12 months.

### Survey questionnaire

The survey questionnaire was divided into two sections: (1) demographic and professional variables and (2) the KAP questionnaire (Supplementary Material 1). The demographic section captured characteristics of the HCPs, including specialties, years of clinical practice, practice settings (public/private/mixed), average number of patients with diabetes managed monthly and experience recommending newly introduced CGM devices. The KAP questionnaire was developed based on existing literature and pertinent clinical practice guidelines on glucose monitoring and diabetes management. An expert panel thoroughly reviewed the questionnaire to ensure its content validity, accuracy and clinical appropriateness. The finalized questionnaire was structured into three domains: knowledge, attitude and practice, with six questions in each domain. Responses were measured using a 5-point Likert scale that spans from ‘5 – strongly agree/highly confident,’ ‘4 – agree/confident,’ ‘3 – neutral,’ ‘2 – disagree/slightly confident’ and ‘1 – strongly disagree/not confident.’

### Data analysis

Descriptive statistics were used to summarize survey responses. Continuous variables were presented as mean ± standard deviation and categorical variables as frequencies and percentages. Descriptive analysis of Likert-scale responses was performed to evaluate the distribution of responses across the KAP domains.

### Ethical considerations

The study adhered to the ethical principles outlined in the Helsinki Declaration of 1964 and its later amendments. All participants provided informed consent before participating, affirming their voluntary involvement and understanding of the study’s purpose and procedures. To ensure participants’ anonymity, no identifying personal information was collected. Since the study did not include any patient data, ethics committee approval was not required.

**Table 1:** Demographic characteristics of study participants

Parameters	HCPs (N = 126)
**Gender, n (%)**	
Male	108 (85.70%)
Female	18 (14.20%)
**Specialty distribution, n (%)**	
Endocrinologists/diabetologists	89 (70.60%)
General/primary care physicians	32 (25.40%)
Other specialists	5 (4.00%)
**Years of clinical experience, n (%)**	
0-5 years	7 (5.50%)
6-10 years	14 (11.10%)
11-15 years	20 (15.90%)
16-20 years	21 (16.70%)
>20 years	64 (50.80%)
**Practice settings, n (%)**	
Government	14 (11.10%)
Private	89 (70.60%)
Institutional	17 (13.50%)
Government and private practice	1 (0.79%)
**Average number of patients with diabetes managed per month, n (%)**	
<100	29 (23.00%)
100-200	39 (31.00%)
200-500	38 (30.20%)
>500	20 (15.90%)
Recommendation of the use of the new FSL2+ CGM* launched in August 2025, n (%)	
Yes	98 (77.80%)
No	28 (22.20%)

*FSL2+ CGM: FreeStyle Libre 2 Plus continuous glucose monitoring system, an advanced wearable medical device launched by Abbott (Abbott Park, IL, USA) CGM = continuous glucose monitoring; FSL2 = FreeStyle Libre 2; HCPs = healthcare professionals; SD = standard deviation.

## Results

### Demographic characteristics of participants

Of the 150 HCPs invited, 126 participated in the study, corresponding to an 84.0% response rate. The participating HCPs were from different specialties, predominantly endocrinologists/diabetologists (70.6%), and practiced in private or public settings in India. The majority of HCPs (50.8%) had more than 20 years of clinical experience (*Table 1*). The distribution of specialists based on clinical experience is shown in Supplementary Table S1.

### Knowledge and clinical confidence in continuous glucose monitoring use and the perceived role of self-monitoring of blood glucose

Most HCPs expressed confidence in the use of CGM for GDM, with 81.7% (103/126) reporting confidence and/or high confidence, whereas only 6.34% (8/126) reported low and/or no confidence. Regarding CGM use in pediatric patients (>2 years), 62.7% (79/126) of HCPs reported confidence and/or high confidence, whereas 18.2 % (23/126) reported low and/or no confidence (*Figure 1a*).

A majority of HCPs (80.1%, 101/126) were either confident or highly confident in interpreting CGM reports, and a comparable proportion (81.8%, 103/126) were confident or highly confident in explaining CGM reports to their patients. Approximately two-thirds of participants (68.2%, 86/126) reported being confident or highly confident in the recommended calibration frequency for CGM devices (*Figure 1a*).

Nonetheless, most HCPs (77.0%, 97/126) agreed or strongly agreed that SMBG remains an important glucose monitoring method despite the availability of CGM (*Figure 1b*).

A subgroup analysis by specialty was conducted to capture HCP responses across specialties regarding their knowledge of CGM. Endocrinologists/diabetologists (n=89) showed favorable perceptions of CGM use (84.3%, 75/89) and higher confidence in interpreting CGM reports (87.6%, 78/89) and explaining them to patients (88.8%, 79/89), compared with other specialists and general/primary care physicians (Table S2).

### Attitudes toward clinical utility, comparative effectiveness and barriers to continuous glucose monitoring use

More than 90% of HCPs (90.5%, 114/126) agreed or strongly agreed that CGM improves overall diabetes management, whereas only 3.2% (4/126) completely disagreed. About four-fifths of participants (77.8%, 98/126) believed that CGM should be used for glucose monitoring in all patients treated with insulin. Moreover, 87.3% of HCPs (110/126) agreed or strongly agreed that CGM functions as a motivational tool and helps them better understand patients’ lifestyles.

The vast majority of HCPs (88.1%, 111/126) were confident and/or highly confident that the use of CGM can help lower hypoglycemic and hyperglycemic episodes compared with SMBG alone. However, 31.7% (40/126) agreed or strongly agreed that CGM can completely replace SMBG, whereas 19.1% (24/126) disagreed or strongly disagreed. Notably, a large majority of HCPs (78.6%, 99/126) agreed or strongly agreed that the cost of CGM represents a major barrier to its wider adoption in diabetes care. *Figure 2* depicts HCPs’ attitudes toward CGM use and the barriers to its use. A subgroup analysis by specialty was conducted to assess differences in HCPs’ attitudes toward CGM and SMBG across specialties. Positive attitudes toward CGM were evident among the surveyed HCPs, with 89.9% of endocrinologists/diabetologists, 93.8% of general/primary care physicians and 80.0% of other specialists agreeing that CGM enhances overall diabetes management. However, agreement that CGM can completely replace SMBG was relatively limited across these groups, particularly among endocrinologists/diabetologists (25.8%), general/primary care physicians (46.9%) and other specialists (40.0%) (Table S3).

### Current clinical practices in continuous glucose monitoring recommendation, data utilization and self-monitoring of blood glucose training

When asked about recommending CGM for glucose monitoring to patients with diabetes, 47.6% (60/126) of HCPs reported recommending CGM, whereas 44.4% (56/126) remained neutral. Moreover, most HCPs (79.3%, 100/126) agreed or strongly agreed that they provide training to their patients on the use of SMBG devices.

A large majority of HCPs (82.53%, 104/126) reported reviewing their patients' CGM reports during follow-up visits, and a similar proportion (82.60%, 104/126) also reported discussing TIR metrics with their patients. Moreover, most HCPs (81.7%, 103/126) agreed or strongly agreed that they use CGM reports to counsel patients on lifestyle modifications, and HCPs (82.53%, 104/126) reported using CGM data to modify treatment regimens (*Figure 3*).

**Figure 1:**
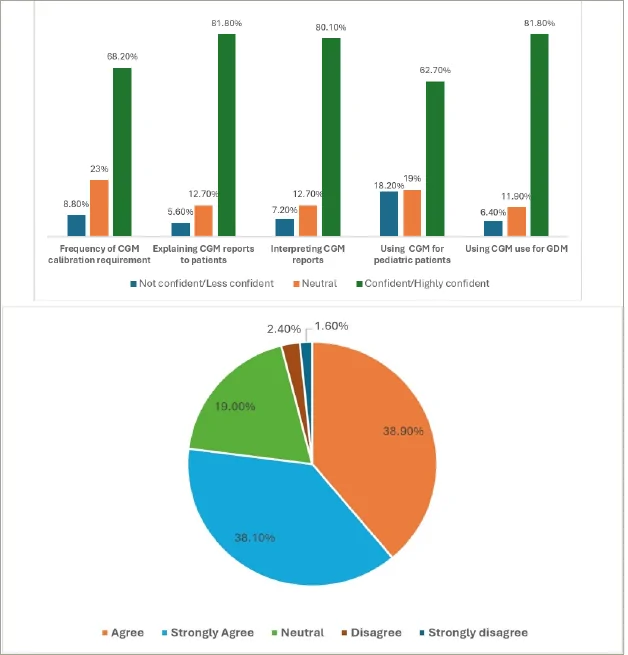
Knowledge of healthcare professionals about continuous glucose monitoring use

*a: Confidence of healthcare professionals about continuous glucose monitoring interpretation and use in diabetes care; b: Perceptions of healthcare professionals about the importance of self-monitoring of blood glucose in the continuous glucose monitoring era. Figure was created using Microsoft Excel (Microsoft, Redmond, WA, USA) CGM = continuous glucose monitoring; GDM = gestational diabetes mellitus*

These findings indicate a high level of knowledge and favorable attitudes among HCPs toward CGM. However, routine clinical recommendations were lower, underscoring an adoption–action gap in CGM use in clinical practice. Responses were also stratified based on the specialties of the HCPs who participated in the survey. Most respondents in these larger groups also reported reviewing CGM reports, discussing TIR, using CGM for lifestyle counseling and modifying treatment based on CGM data. Detailed findings are presented in Table S4. The knowledge, attitudes and clinical practices related to CGM use have been summarized in *Figure 4*.

## Discussion

The current study provides a contemporary assessment of knowledge, attitudes and reported clinical practices regarding CGM use in diabetes care among HCPs from diverse specialties and practice settings across India. Notably, the majority of the surveyed HCPs were diabetologists and endocrinologists with more than 20 years of clinical experience, primarily practicing in private healthcare settings. A key finding of the study is the high level of knowledge, positive attitudes and perceived clinical value of CGM among surveyed HCPs, indicating substantial awareness of its role in diabetes management. A favorable perception of CGM was observed across the larger specialty groups, although support for completely replacing SMBG was limited. Despite high levels of knowledge and positive attitudes toward CGM, a gap exists between perceptions and their translation into routine clinical practice, highlighting an adoption–action gap. Cost was identified as the key barrier to CGM adoption in diabetes care in India.

The majority of participating HCPs reported confidence in interpreting CGM reports, communicating results to patients and explaining CGM metrics, such as TIR. However, subgroup analysis indicated greater confidence among diabetologists/endocrinologists than among general/primary care physicians and other specialists in interpreting CGM reports and explaining the results to their patients. This finding about knowledge of and confidence in CGM across HCP groups is consistent with the increasing integration of CGM into clinical practice guidelines and diabetes education. Current clinical practice guidelines from the American Diabetes Association (ADA) and consensus recommendations from the Research Society for the Study of Diabetes in India (RSSDI)–Endocrine Society of India highlight the importance of TIR, TAR and TBR in complementing HbA1c for assessing glycemic control.^9,14^ These CGM parameters provide a comprehensive picture of individuals’ glycemic patterns and variability that HbA1c alone cannot capture.^15^ Real-world evidence from India supports the clinical utility of CGM-derived metrics; a multicenter study across 7 Indian diabetes clinics involving 5,072 patients with diabetes (T1DM or T2DM) demonstrated that flash glucose monitoring-based ambulatory glucose profiling was associated with a greater reduction in HbA1c than in matched controls.^16^ In another single-center real-world study from India involving 649 adults with T2DM, TIR was significantly negatively associated with lower HbA1c, mean glucose and TAR and positively associated with TBR, supporting the clinical utility of CGM metrics for glycemic control and treatment optimization.^17^ Hence, integrating CGM metrics with HbA1c offers a more holistic and clinically valuable approach to improve glycemic assessment and personalized therapeutic adjustments than relying on either measure alone.^1,8,18^

**Figure 2:**
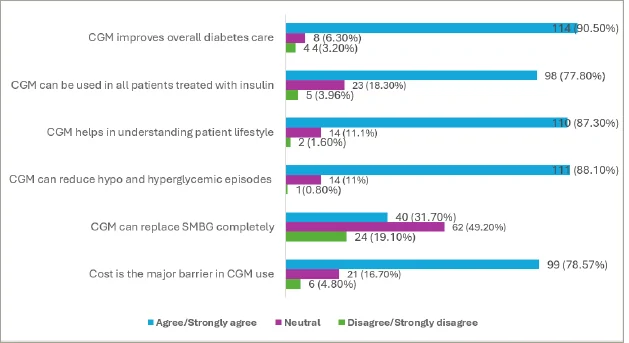
Attitudes of healthcare professionals toward continuous glucose monitoring and barriers to its use among patients with diabetes

*Figure was created using Microsoft Excel (Microsoft, Redmond, WA, USA) CGM = continuous glucose monitoring; SMBG = self-monitoring of blood glucose*

In addition to its use in T1DM and T2DM, the ADA and RSSDI practice guidelines recommend CGM for pregnant women with GDM.^9,14^ Accordingly, many participating HCPs reported being confident in recommending CGM for patients with GDM, suggesting familiarity with CGM among the surveyed HCPs. However, HCPs in this study reported lower confidence in recommending CGM for pediatric patients (>2 years of age), indicating that additional training and clinical experience with CGM technologies may be beneficial.

Despite awareness of CGM’s clinical value, many participating HCPs continued to consider SMBG an essential glucose monitoring method in the CGM era and reported training patients to use SMBG devices. This perception likely reflects the widespread acceptance of SMBG by both HCPs and patients for more than five decades.^19^ This is especially applicable in resource-limited settings, where SMBG remains a standard and practical option, given the limited CGM availability and accessibility.^20^ Fewer than 50% of HCPs in our study believed that CGM could completely replace SMBG, whereas nearly half remained neutral. This finding indicates that although CGM can provide more comprehensive insights into glycemic trends than conventional SMBG, it is still not widely perceived as a complete replacement for SMBG. Several limitations, such as the higher cost of CGM devices, discrepancies between CGM and glucometer readings (especially during major glycemic fluctuations), shorter sensor wear time and patients’ educational levels, learning capacities and technological literacy, may contribute to the surveyed HCPs’ perceptions regarding CGM use. Therefore, SMBG and CGM appear to be complementary tools that align with patient-centered care for effective diabetes management.^20^

**Figure 3:**
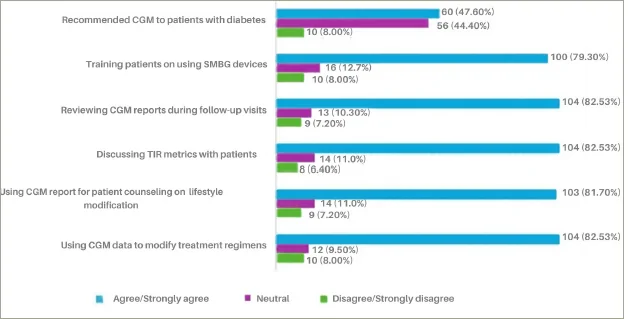
Healthcare professional perceptions of continuous glucose monitoring use in clinical practice

*Healthcare professionals indicated their level of agreement (4 or 5) on a 5-point Likert scale (1 – Strongly disagree; 5 – Strongly agree). Figure was created using Microsoft Excel (Microsoft, Redmond, WA, USA)*

*CGM = continuous glucose monitoring; SMBG = self-monitoring of blood glucose; TIR = time in range.*

**Figure 4:**
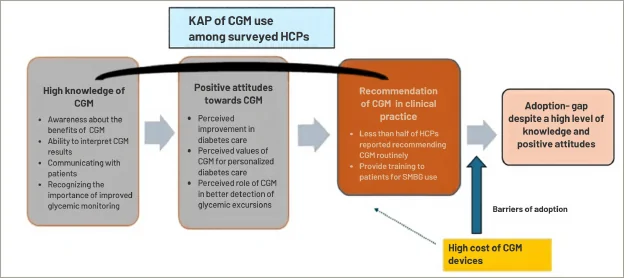
Knowledge, attitudes and clinical practices related to continuous glucose monitoring use

*Figure was created using Microsoft PowerPoint (Microsoft, Redmond, WA, USA)*

*CGM = continuous glucose monitoring; HCPs = healthcare professionals; KAP = knowledge, attitudes and practices; SMBG = self-monitoring of blood glucose.*

Another notable finding was that although most participating HCPs acknowledged the benefits of CGM for diabetes management and reducing GV, fewer than 50% routinely recommended CGM to their patients, whereas nearly 50% (especially endocrinologists/diabetologists) reported a neutral response, highlighting a gap between clinical perceptions and reported practice. Likewise, a previous study reported that clinicians often hesitate to recommend CGM when most patients resist it, insurance reimbursement is limited and additional time and effort are required for device support.^21^ The adoption–action gap may reflect systemic and economic barriers, including limited access to CGM devices, lack of reimbursement, high out-of-pocket costs, urban–rural disparities in awareness, insufficient HCP training and education, limited access to technologies, affordability issues and lack of support in resource-constrained settings.^12,21–23^ In our study, cost was the most frequently reported barrier to CGM use. Consistent with this finding, the high cost of CGM devices and limited insurance coverage have been identified as important barriers to CGM adoption in LMICs.^24^ Affordability challenges have been noted across LMICs, where a month’s supply of CGM/flash sensors was estimated to cost 15–65 days’ wages, indicating considerable financial barriers to access in resource-constrained settings.^25^ Furthermore, a US-based study reported that despite broad HCP support for CGM, major barriers to its use included cost, insurance coverage and difficulties obtaining prescriptions.^26^

A vast majority of surveyed HCPs, especially diabetologists/endocrinologists, perceived CGM as a motivational tool and a means of understanding patients’ lifestyles, in addition to its role in glycemic control. This perception is supported by evidence suggesting that CGM’s visual feedback can help promote proactive behavioral changes. More importantly, CGM serves as an empowering tool for patients with diabetes, strengthening self-management, improving abnormal GV and motivating favorable lifestyle behaviors, such as physical activity and dietary changes.^27^ Moreover, the real-t ime education provided by CGM, which delivers personalized insights into each patient’s unique physiological responses, is likely to be more effective for glucose monitoring and management than traditional methods.^28^ By providing continuous glucose profiles with point-in-time glucose values and a trend arrow indicating a rise or fall, CGM allows HCPs to better understand how lifestyle choices, dietary patterns, physical activity and medications affect patients in real time, thereby supporting informed daily diabetes management.^29,30^

The findings of our study revealed that while the participating HCPs understand the importance of CGM and are aware of its use, broader implementation remains challenging in India. Bridging the gap between knowledge and clinical adoption is crucial to enhancing CGM access in diabetes care. This requires a comprehensive approach involving systemic, educational and economic measures, such as increasing patient awareness, improving HCP training in CGM interpretation, integrating CGM data into electronic health records, advancing technology, expanding insurance coverage, improving the affordability of CGM devices and developing India-specific clinical guidelines.^12,20,31^

The study has several limitations. The cross-sectional design limits the ability to establish causal relationships between knowledge and clinical practice. The relatively small sample size and predominance of experienced endocrinologists/diabetologists from the private sector may limit the generalizability of the findings, particularly to family physicians, general practitioners and other HCPs involved in diabetes care in India. As with other survey-based research, the findings may be influenced by recall, response and social desirability biases due to self-reported data. Moreover, purposive sampling via online medical panels and conference attendees may have introduced selection bias, as HCPs with a greater interest in diabetes care or CGM use may have been more likely to participate. Furthermore, since patient-level utilization rates and objective measures of CGM and SMBG adoption were not evaluated, the study could not assess the proportion of patients using these technologies, their direct clinical impact in routine practice or whether reported practices translate into real-world adoption. Finally, patient-level costs, healthcare utilization and monitoring frequency were not evaluated; therefore, the comparative cost-effectiveness of CGM versus SMBG could not be assessed.

The key strength of our study is its assessment of KAP regarding CGM use among HCPs across multiple specialties in India, providing diverse perspectives on its use in routine clinical practice.

### Implications for policy and future research

The findings of this study highlight opportunities to strengthen HCP education and training in CGM interpretation and clinical application, as well as to consider CGM in relevant clinical guidelines. Moreover, improving affordability and reimbursement strategies through greater recognition by regulators and payers may support the appropriate use of CGM in clinical practice. Future research should include larger and more representative samples of HCPs, as well as longitudinal studies to assess changes in CGM adoption and practice. Additionally, future studies assessing patient perspectives, affordability, patient-level outcomes and healthcare resource utilization are needed to inform the appropriate integration of CGM into diabetes care in India.

## Conclusion

The study demonstrates that HCPs across India have a strong awareness and appreciation of CGM’s clinical value in diabetes care. However, cost constraints, variability in confidence in CGM interpretation and greater reliance on the conventional SMBG method contribute to an adoption–action gap. Addressing structural and economic barriers, along with strengthening practical training, may facilitate broader implementation of CGM and support personalized diabetes care.

## Supplementary Materials

Supplementary files
