# Supplementary material for: Knowledge, attitude and practice on continuous glucose monitoring and self-monitoring of blood glucose among healthcare professionals across India: A cross-sectional study
Source: touchREV Endocrinol. 2026 Oct 20;22(2):48–54. doi: 10.17925/EE.2026.22.2.7 (PMC13730174; doi:10.17925/EE.2026.22.2.7)
Supplement: Supplementary file 1 [file touchendo-22-2-48_supp.pdf]

Supplementary files

KAP survey questionnaire

Knowledge, Attitude, and Practice (KAP) Questionnaire on Continuous Glucose Monitoring (CGM) and Self-Monitoring of Blood Glucose (SMBG)

1. Disclaimer

Check all that apply.

☐ Participation in this survey is completely voluntary. The responses collected will be used solely for academic and research purposes to understand diabetologists' perspectives and practices related to diabetes management. No personal identifiers will be collected, and all responses will remain strictly confidential. By proceeding, you consent to the use of your anonymized data for analysis and publication in aggregated form.

2. Email *

3. Name*

4. Location*

1. Profession:*

Mark only one oval.

○ Physician

○ Diabetologist

○ Endocrinologist

○ Other:

2. Years of clinical experience:*

Mark only one oval.

○ 0-5

○ 6-10

○ 11-15

○ 16-20

○ >20

3. Type of practice:*

Mark only one oval.

- ○ Government
- ○ Private
- ○ Institutional
- ○ Other:

4. Average number of diabetic patients managed per month: *

Mark only one oval.

- ○ <100
- ○ 100-200
- ○ 200-500
- ○ >500

5. Recommending the uses of the new FSL2+ CGM launched in July'25:*

Mark only one oval.

- ○ Yes
- ○ No

Knowledge

Please indicate your level of agreement using the following scale:

1 = No Confidence, 2 = Low Confidence, 3 = Neutral, 4 = Confident, 5 = Highly Confident

1. How confident are you with the use of CGM for GDM indications? *

Mark only one oval

1	2	3	4	5
○	○	○	○	○

2. How confident are you with the use of CGM for pediatric (> 2 years) indications? *

Mark only one oval

1	2	3	4	5
○	○	○	○	○

3. Your confidence in interpreting CGM reports? *

Mark only one oval

1	2	3	4	5
⬭	⬭	⬭	⬭	⬭

4. How confident are you with explaining CGM reports to patients? *

Mark only one oval

1	2	3	4	5
⬭	⬭	⬭	⬭	⬭

5. How confident are you with the frequency of calibration required for CGM devices?

Mark only one oval

1	2	3	4	5
⬭	⬭	⬭	⬭	⬭

6. SMBG remains important even when CGM is available. *

Mark only one oval

1	2	3	4	5
⬭	⬭	⬭	⬭	⬭

Attitude

Please indicate your level of agreement using the following scale:

1 = Strongly Disagree, 2 = Disagree, 3 = Neutral, 4 = Agree, 5 = Strongly Agree

1. CGM improves overall diabetes management. *

Mark only one oval

1	2	3	4	5
⬭	⬭	⬭	⬭	⬭

2. CGM should be used in all insulin-treated patients. *

Mark only one oval

1	2	3	4	5

3. CGM data helps in a better understanding of patient lifestyles and works as a motivational tool.*

Mark only one oval

1	2	3	4	5

4. How confident are you that use of CGM can help reduce hypoglycemic and hyperglycemic episodes compared to SMBG alone?*

Mark only one oval

1	2	3	4	5

5. I believe CGM can replace SMBG completely. *

Mark only one oval

1	2	3	4	5

6. The cost of CGM is a major barrier to its use. *

Mark only one oval

1	2	3	4	5

Practice

Please indicate how often you perform the following activities:

1 = Never, 2 = Rarely, 3 = Sometimes, 4 = Often, 5 = Always 22. Mark only one oval

1. I recommend CGM to my patients with diabetes. *

Mark only one oval

1	2	3	4	5
◯	◯	◯	◯	◯

2. I train patients on how to use SMBG devices. *

Mark only one oval

1	2	3	4	5
◯	◯	◯	◯	◯

3. I personally review CGM reports during follow-up visits. *

Mark only one oval

1	2	3	4	5
◯	◯	◯	◯	◯

4. I discuss 'Time in Range' metrics with patients. *

Mark only one oval

1	2	3	4	5
◯	◯	◯	◯	◯

5. I use CGM reports to counsel patients on lifestyle modification. *

Mark only one oval

1	2	3	4	5
◯	◯	◯	◯	◯

6. I use CGM data to modify treatment regimens. *

Mark only one oval

1	2	3	4	5
◯	◯	◯	◯	◯

Supplementary Tables

Supplementary Table 1: Distribution of specialists based on years of clinical experience

Years of clinical experience	Endocrinologist / Diabetologist	General / Primary Care Physicians	Other Specialists	Total
**0–5 years**	5 (5.6%)	2 (6.3%)	0 (0.0%)	7 (5.6%)
**6–10 years**	12 (13.5%)	2 (6.3%)	0 (0.0%)	14 (11.1%)
**11–15 years**	13 (14.6%)	6 (18.8%)	1 (20.0%)	20 (15.9%)
**16–20 years**	18 (20.2%)	2 (6.3%)	1 (20.0%)	21 (16.7%)
**>20 years**	41 (46.1%)	20 (62.5%)	3 (60.0%)	64 (50.8%)
**Total**	**89 (100.0%)**	**32 (100.0%)**	**5 (100.0%)**	**126 (100.0%)**

Supplementary Table 2: Subgroup analysis of knowledge level based on different specialists' responses

Questions	Response	Endocrinologists / Diabetologists (n=89)	General / Primary Care Physicians (n=32)	Other Specialists (n=5)	Total (N=126)
**How confident are you with the use of CGM for GDM indications?**	Disagree	3 (3.4%)	4 (12.5%)	1 (20.0%)	8 (6.3%)
	Neutral	11 (12.4%)	2 (6.3%)	2 (40.0%)	15 (11.9%)
	Agree	75 (84.3%)	26 (81.3%)	2 (40.0%)	103 (81.7%)
**How confident are you with the use of CGM for pediatric (>2 years) indications?**	Disagree	8 (9.0%)	13 (40.6%)	2 (40.0%)	23 (18.3%)
	Neutral	16 (18.0%)	7 (21.9%)	1 (20.0%)	24 (19.0%)
	Agree	65 (73.0%)	12 (37.5%)	2 (40.0%)	79 (62.7%)
**Your confidence in interpreting CGM reports**	Disagree	4 (4.5%)	4 (12.5%)	1 (20.0%)	9 (7.1%)
	Neutral	7 (7.9%)	6 (18.8%)	3 (60.0%)	16 (12.7%)
	Agree	78 (87.6%)	22 (68.8%)	1 (20.0%)	101 (80.2%)
**How confident are you with explaining CGM**	Disagree	2 (2.2%)	4 (12.5%)	1 (20.0%)	7 (5.6%)
	Neutral	8 (9.0%)	5 (15.6%)	3 (60.0%)	16 (12.7%)

**reports to patients?**	Agree	79 (88.8%)	23 (71.9%)	1 (20.0%)	103 (81.7%)
**How confident are you with the frequency of calibration required for CGM devices?**	Disagree	6 (6.7%)	4 (12.5%)	1 (20.0%)	11 (8.7%)
	Neutral	15 (16.9%)	10 (31.3%)	4 (80.0%)	29 (23.0%)
	Agree	68 (76.4%)	18 (56.3%)	0 (0.0%)	86 (68.3%)
**SMBG remains important even when CGM is available.**	Disagree	3 (3.4%)	1 (3.1%)	1 (20.0%)	5 (4.0%)
	Neutral	17 (19.1%)	6 (18.8%)	1 (20.0%)	24 (19.0%)
	Agree	69 (77.5%)	25 (78.1%)	3 (60.0%)	97 (77.0%)

Disagree = responses 1–2; Neutral = response 3; Agree = responses 4–5.Percentages are calculated within each professional specialty group.

Supplementary Table 3: Subgroup analysis of CGM and SMBG attitude levels based on responses by different HCP specialties

Questions	Response	Endocrinologists / Diabetologists (n=89)	General / Primary Care Physicians (n=32)	Other Specialists (n=5)	Total (N=126)
**1. CGM improves overall diabetes management.**	Disagree	4 (4.5%)	0 (0.0%)	0 (0.0%)	4 (3.2%)
	Neutral	5 (5.6%)	2 (6.3%)	1 (20.0%)	8 (6.3%)
	Agree	80 (89.9%)	30 (93.8%)	4 (80.0%)	114 (90.5%)
**2. CGM should be used in all insulin-treated patients.**	Disagree	4 (4.5%)	0 (0.0%)	1 (20.0%)	5 (4.0%)
	Neutral	17 (19.1%)	4 (12.5%)	2 (40.0%)	23 (18.3%)
	Agree	68 (76.4%)	28 (87.5%)	2 (40.0%)	98 (77.8%)
**3. CGM data helps in a better understanding of patient lifestyles and works as a motivational tool.**	Disagree	2 (2.2%)	0 (0.0%)	0 (0.0%)	2 (1.6%)
	Neutral	9 (10.1%)	3 (9.4%)	2 (40.0%)	14 (11.1%)
	Agree	78 (87.6%)	29 (90.6%)	3 (60.0%)	110 (87.3%)
**4. How confident are you that use of CGM can help reduce hypoglycemic and hyperglycemic episodes compared to SMBG alone?**	Disagree	1 (1.1%)	0 (0.0%)	0 (0.0%)	1 (0.8%)
	Neutral	10 (11.2%)	3 (9.4%)	1 (20.0%)	14 (11.1%)
	Agree	78 (87.6%)	29 (90.6%)	4 (80.0%)	111 (88.1%)
**5. I believe CGM can replace SMBG completely.**	Disagree	19 (21.3%)	5 (15.6%)	0 (0.0%)	24 (19.0%)
	Neutral	47 (52.8%)	12 (37.5%)	3 (60.0%)	62 (49.2%)

	Agree	23 (25.8%)	15 (46.9%)	2 (40.0%)	40 (31.7%)
**6. The cost of CGM is a major barrier to its use.**	Disagree	4 (4.5%)	2 (6.3%)	0 (0.0%)	6 (4.8%)
	Neutral	13 (14.6%)	7 (21.9%)	1 (20.0%)	21 (16.7%)
	Agree	72 (80.9%)	23 (71.9%)	4 (80.0%)	99 (78.6%)

Disagree = responses 1–2; Neutral = response 3; Agree = responses 4–5. Percentages are calculated within each professional specialty group.

Supplementary Table 4: Subgroup analysis of CGM and SMBG use in clinical practice based on responses of HCP specialties

Questions	Response	Endocrinologists / Diabetologists (n=89)	General / Primary Care Physicians (n=32)	Other Specialists (n=5)	Total (N=126)
**1. I recommend CGM to my patients with diabetes.**	Disagree	5 (5.6%)	2 (6.3%)	3 (60.0%)	10 (7.9%)
	Neutral	42 (47.2%)	12 (37.5%)	2 (40.0%)	56 (44.4%)
	Agree	42 (47.2%)	18 (56.3%)	0 (0.0%)	60 (47.6%)
**2. I train patients on how to use SMBG devices.**	Disagree	5 (5.6%)	3 (9.4%)	2 (40.0%)	10 (7.9%)
	Neutral	10 (11.2%)	5 (15.6%)	1 (20.0%)	16 (12.7%)
	Agree	74 (83.1%)	24 (75.0%)	2 (40.0%)	100 (79.4%)
**3. I personally review CGM**	Disagree	3 (3.4%)	3 (9.4%)	3 (60.0%)	9 (7.1%)
	Neutral	8 (9.0%)	5 (15.6%)	0 (0.0%)	13 (10.3%)

**reports during follow-up visits.**	Agree	78 (87.6%)	24 (75.0%)	2 (40.0%)	104 (82.5%)
**4. I discuss 'Time in Range' metrics with patients.**	Disagree	2 (2.2%)	3 (9.4%)	3 (60.0%)	8 (6.3%)
	Neutral	10 (11.2%)	3 (9.4%)	1 (20.0%)	14 (11.1%)
	Agree	77 (86.5%)	26 (81.3%)	1 (20.0%)	104 (82.5%)
**5. I use CGM reports to counsel patients on lifestyle modification.**	Disagree	3 (3.4%)	3 (9.4%)	3 (60.0%)	9 (7.1%)
	Neutral	10 (11.2%)	4 (12.5%)	0 (0.0%)	14 (11.1%)
	Agree	76 (85.4%)	25 (78.1%)	2 (40.0%)	103 (81.7%)
**6. I use CGM data to modify treatment regimens.**	Disagree	4 (4.5%)	3 (9.4%)	3 (60.0%)	10 (7.9%)
	Neutral	11 (12.4%)	1 (3.1%)	0 (0.0%)	12 (9.5%)
	Agree	74 (83.1%)	28 (87.5%)	2 (40.0%)	

Disagree = responses 1–2; Neutral = response 3; Agree = responses 4–5. Percentages are calculated within each professional specialty group.
